# Supplementary material for: Proteomic Analysis of Rap1A GTPase Signaling-Deficient C57BL/6 Mouse Pancreas and Functional Studies Identify an Essential Role of Rap1A in Pancreas Physiology
Source: Int J Mol Sci. 2024 Jul 23;25(15):8013. doi: 10.3390/ijms25158013 (PMC11312117; doi:10.3390/ijms25158013)
Supplement: Supplementary file 1 [file ijms-25-08013-s001.zip › ijms-3072561-supplementary.pdf]

Supplementary Material

Proteomic Analysis of Rap1A GTPase Signaling-Deficient C57BL/6 Mouse Pancreas and Functional Studies Identify an Essential Role of Rap1A in Pancreas Physiology

Durrey Shahwar, Sadaf Baqai, Faisal Khan, M. Israr Khan, Shafaq Javaid, Abdul Hameed, Aisha Raza, Sadaf Saleem Uddin, Hina Hazrat, M. Hafizur Rahman, Syed Ghulam Musharraf, Maqsood A. Chotani

Supplementary Figure S1: Genotyping

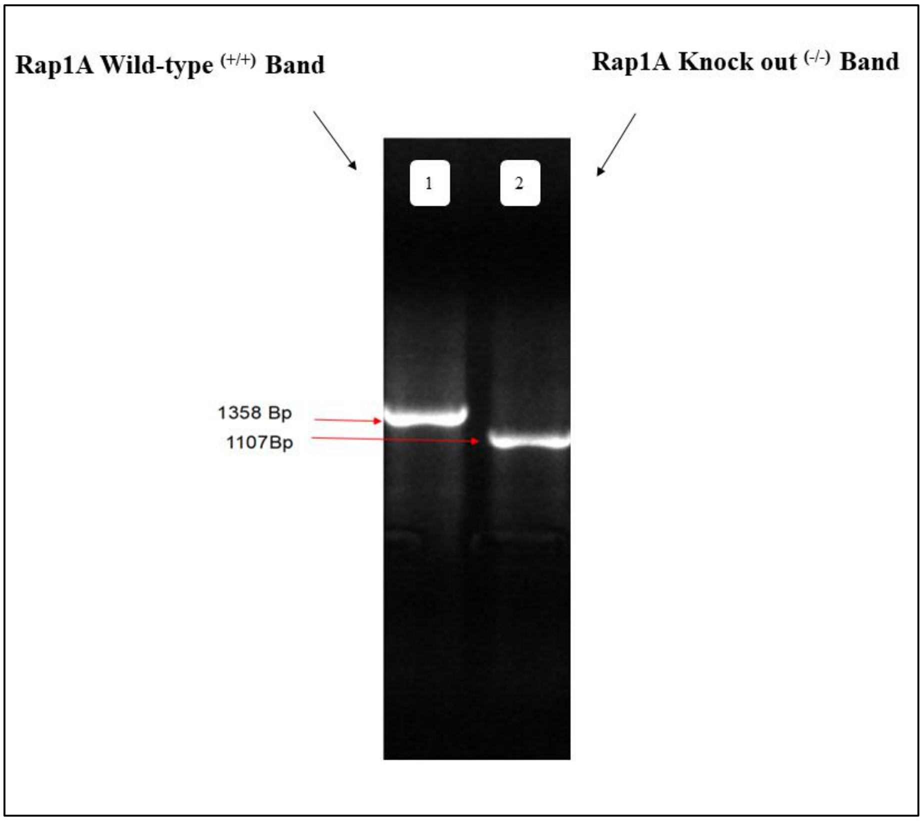

Figure S1: Genotyping Wild-type and Rap1A-Knock-out mice for the study. Lane 1 contains an intact Rap1A exon 4 band of 1,358 bp (wild-type), while Lane 2 contains a Rap1A exon 4 replaced with the neo-gene, band of 1,107 bp (Rap1A-Knock-out).

Supplementary Figure S2: Protein-protein Interaction Analysis

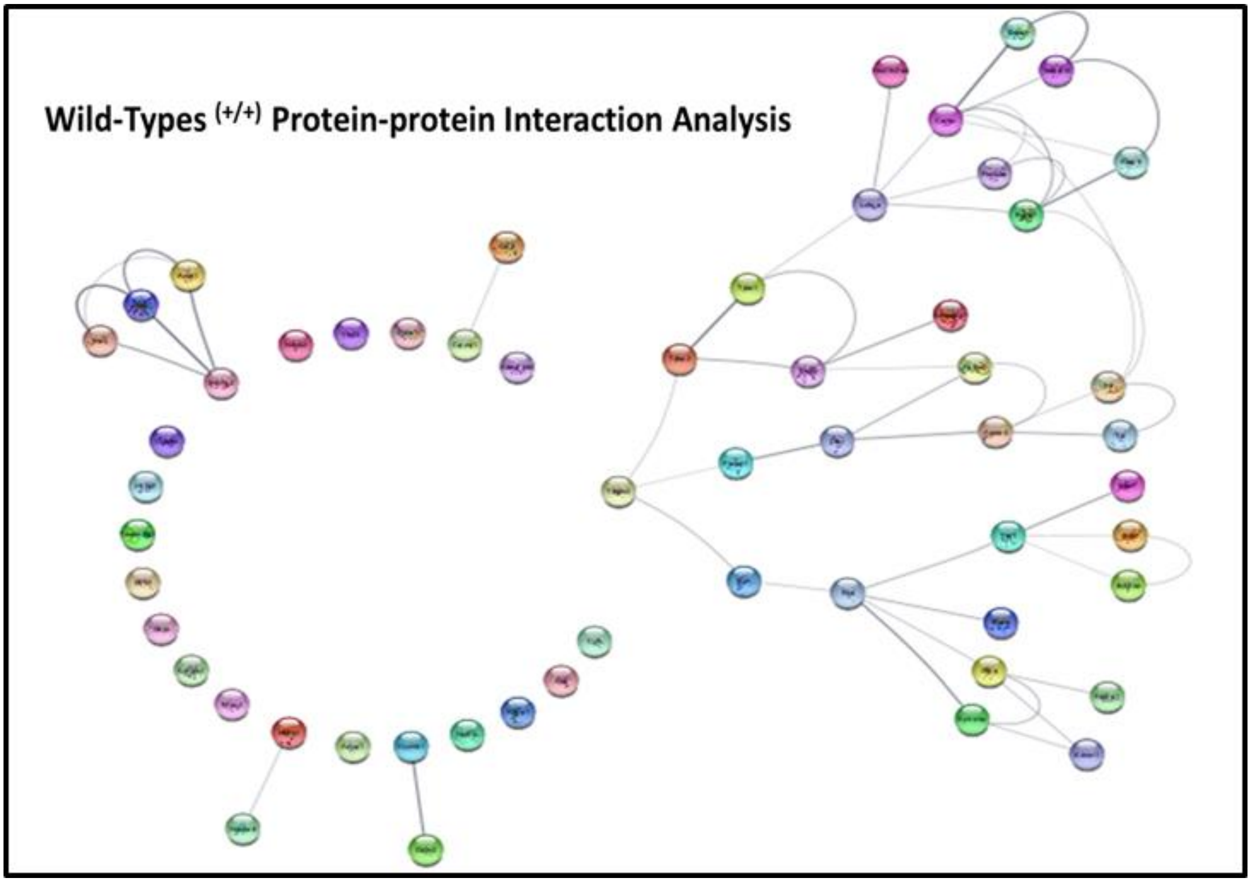

Figure S2: Wild-type sample PPI network. A total of 56 numbers of nodes and 53 PPI network edges with a local clustering coefficient of 0.397.

Supplementary Figure S3: Overlapping Protein-protein Interaction Analysis

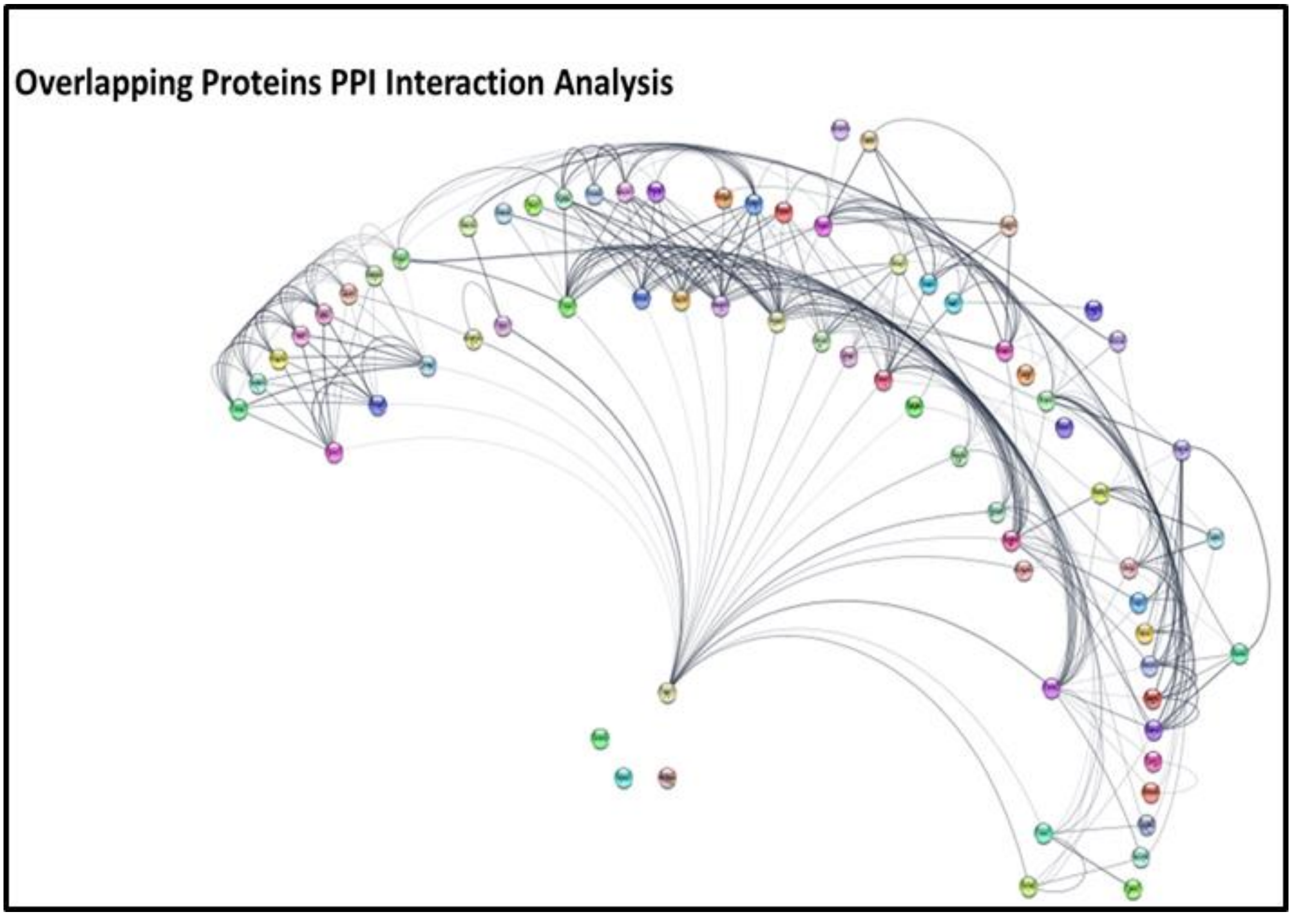

Figure S3: PPI of overlapping proteins. A total of 71 numbers of nodes and 338 PPI network edges with a local clustering coefficient of 0.569.

Supplementary Figures (S4-S9)

KEGG Pathway Analysis of Wild-type $^{(+/+)}$ and Rap1A Knock-out $^{(-/-)}$ Samples (Figures S4 – S9)

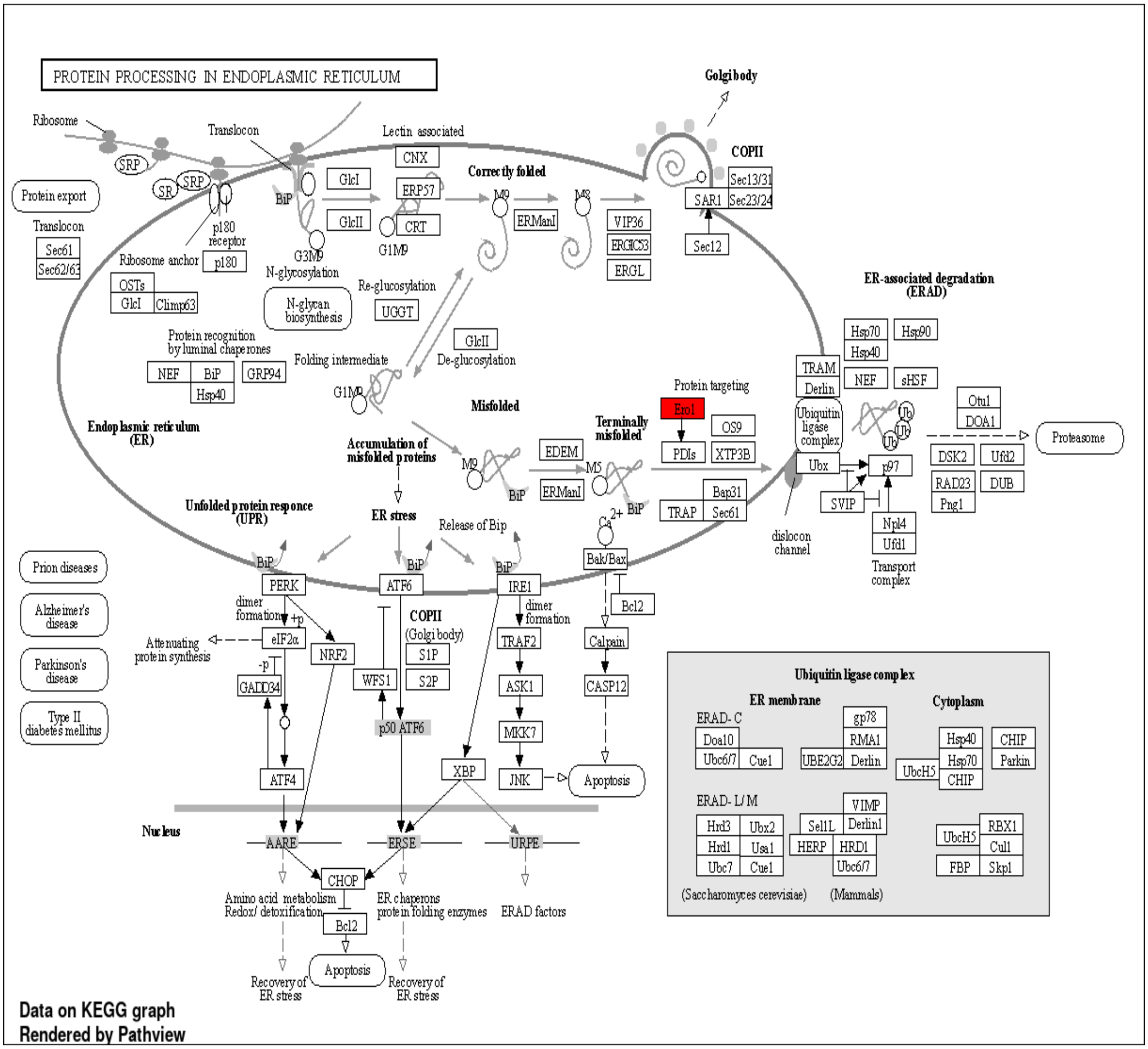

Figure S4: Protein processing in the endoplasmic reticulum, Rap1A knock-out samples. Ero1 representing ERO1 like protein beta (Ero1β), a unique Identifier protein which appears only in Rap1A knock-out $^{(-/-)}$ experimental group.

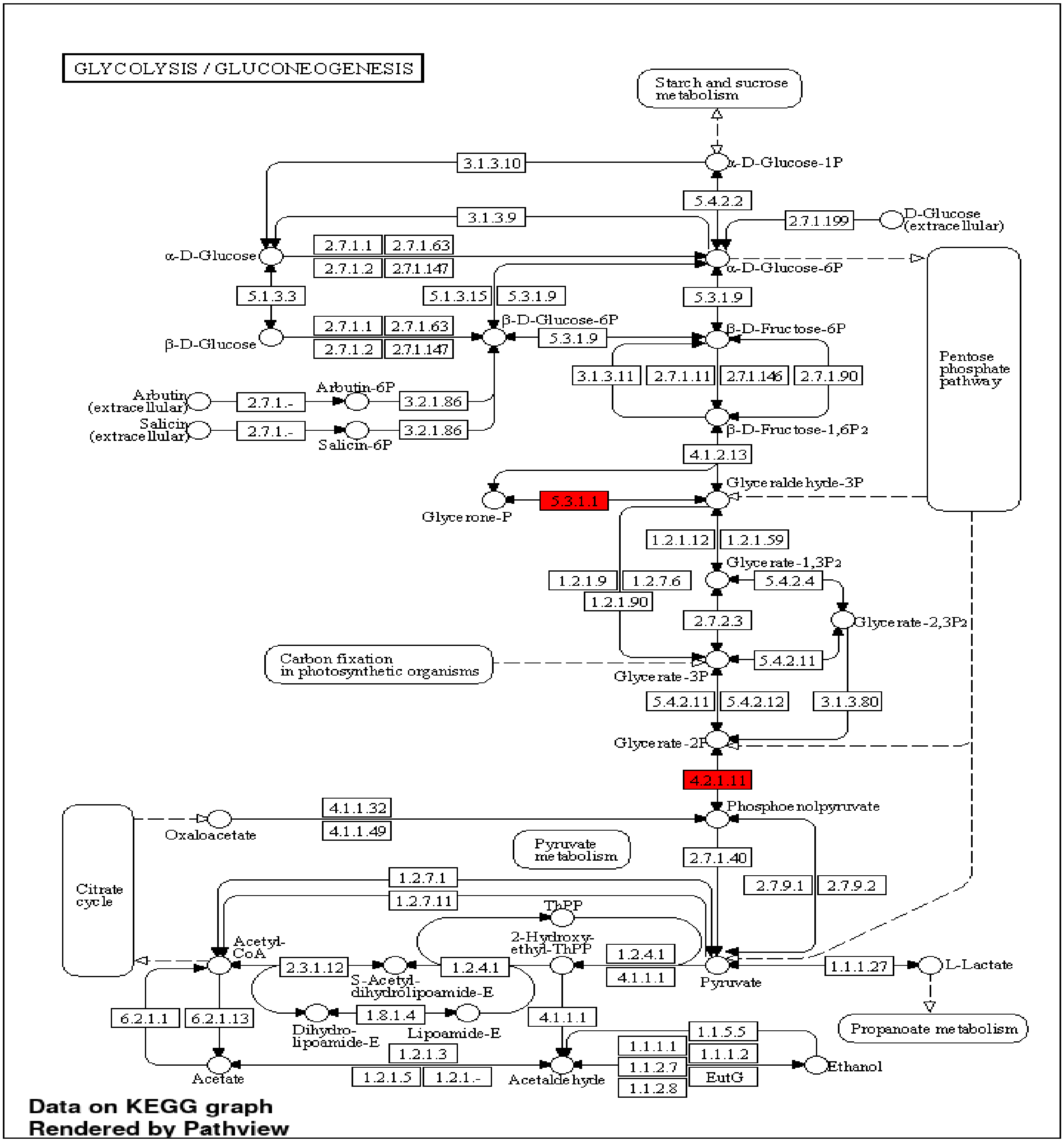

Figure S5: Glycolysis/Gluconeogenesis, Rap1A knock-out samples. 5.3.1.1 representing TP1--Triosephosphate isomerase, a unique identifier protein which appears only in Rap1A knock-out $^{(-/-)}$ sample group.

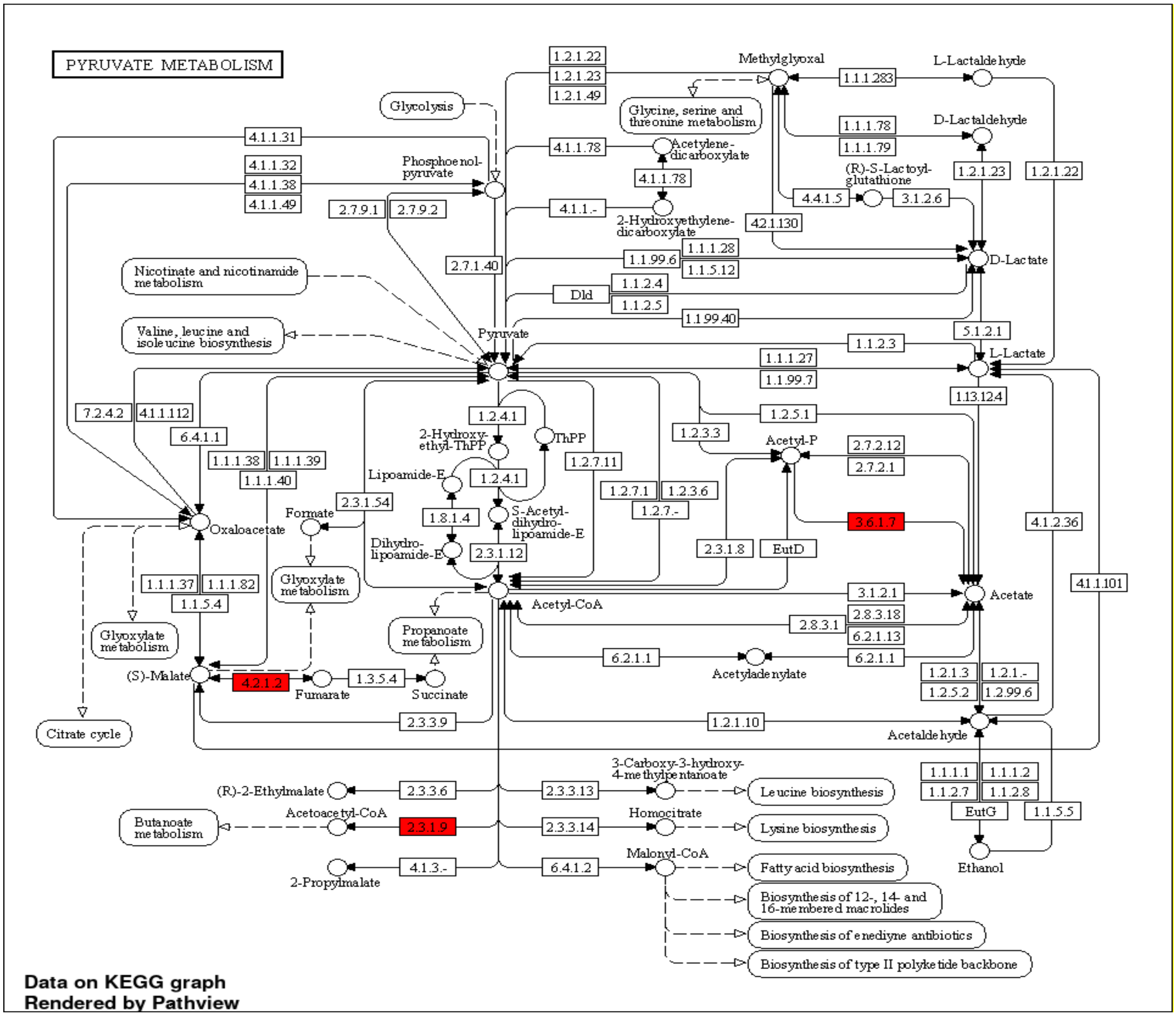

Figure S6: Pyruvate metabolism, Wild-type samples. 4.2.1.2 representing Fh1--- Fumarate hydratase, mitochondria, 2.3.1.9 representing Acat1--- Acetyl-CoA acetyltransferase, mitochondrial, 3.6.1.7 representing Acyp1--- Acylphosphatase-1. All three unique Identifier proteins only appear in Wild-type experimental group.

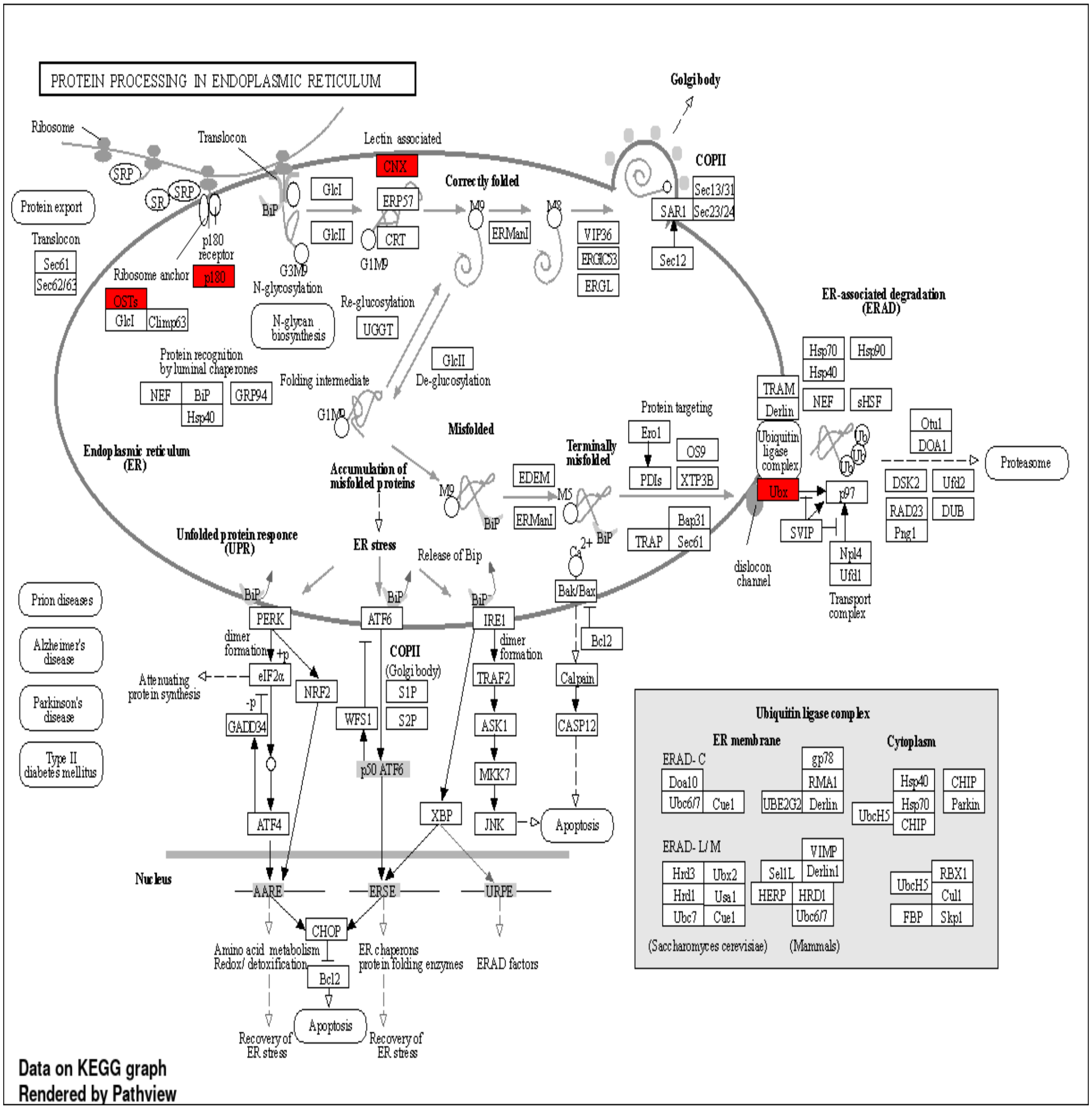

Figure S7: Protein processing in the endoplasmic reticulum, Wild-type samples. UBx an abbreviation for Nsfl1c --- NSFL1 cofactor p47, Canx abbreviation for calnexin, p180 is an abbreviation Rrbp1--- Ribosomebinding protein 1, OSTs is an abbreviation for Ddost --- Dolichyl-diphosphooligosaccharide protein.glycosyltransferase 48 kDa subunit. The highlighted unique identifier proteins are involved in peptide protein folding in endoplasmic reticulum which only appear in Wild-type experimental sample group.

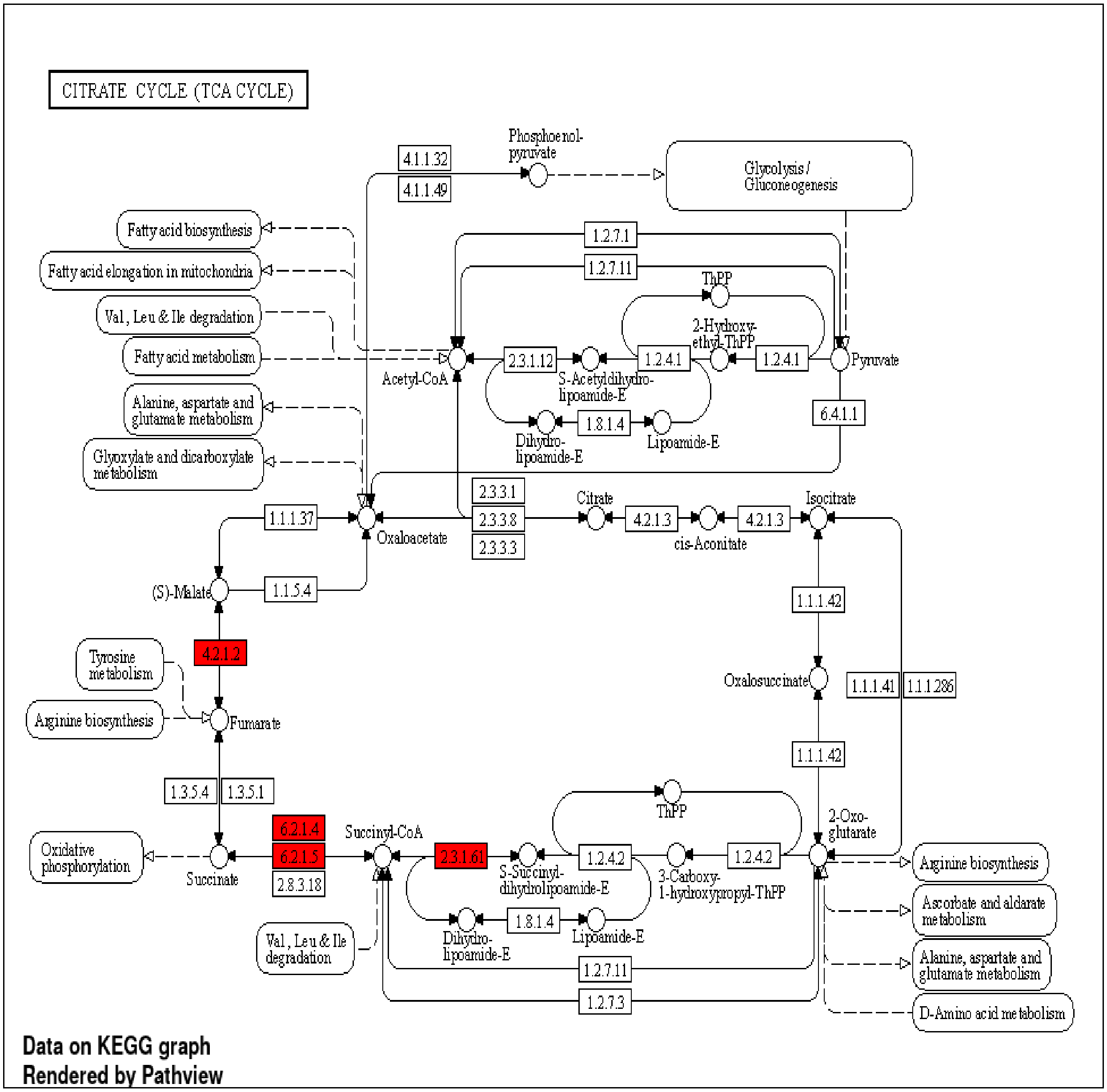

Figure S8: Citrate cycle (TCA Cycle), Wild-type samples. 4.2.1.2 representing fh1--fumarate hydratase, mitochondrial, 6.2.1.4 and 6.2.1.5 representing Suclg2--Succinate--CoA ligase [GDP-forming] subunit β, mitochondrial and 2.3.1.61 representing Dlst---Dihydrolipoyllysine-residue succinyltransferase component of 2-oxoglutarate dehydrogenase complex, mitochondrial. The highlighted unique identifier proteins only appear in Wild-type experimental group and are involved in enzymatic reactions of citric acid cycle.

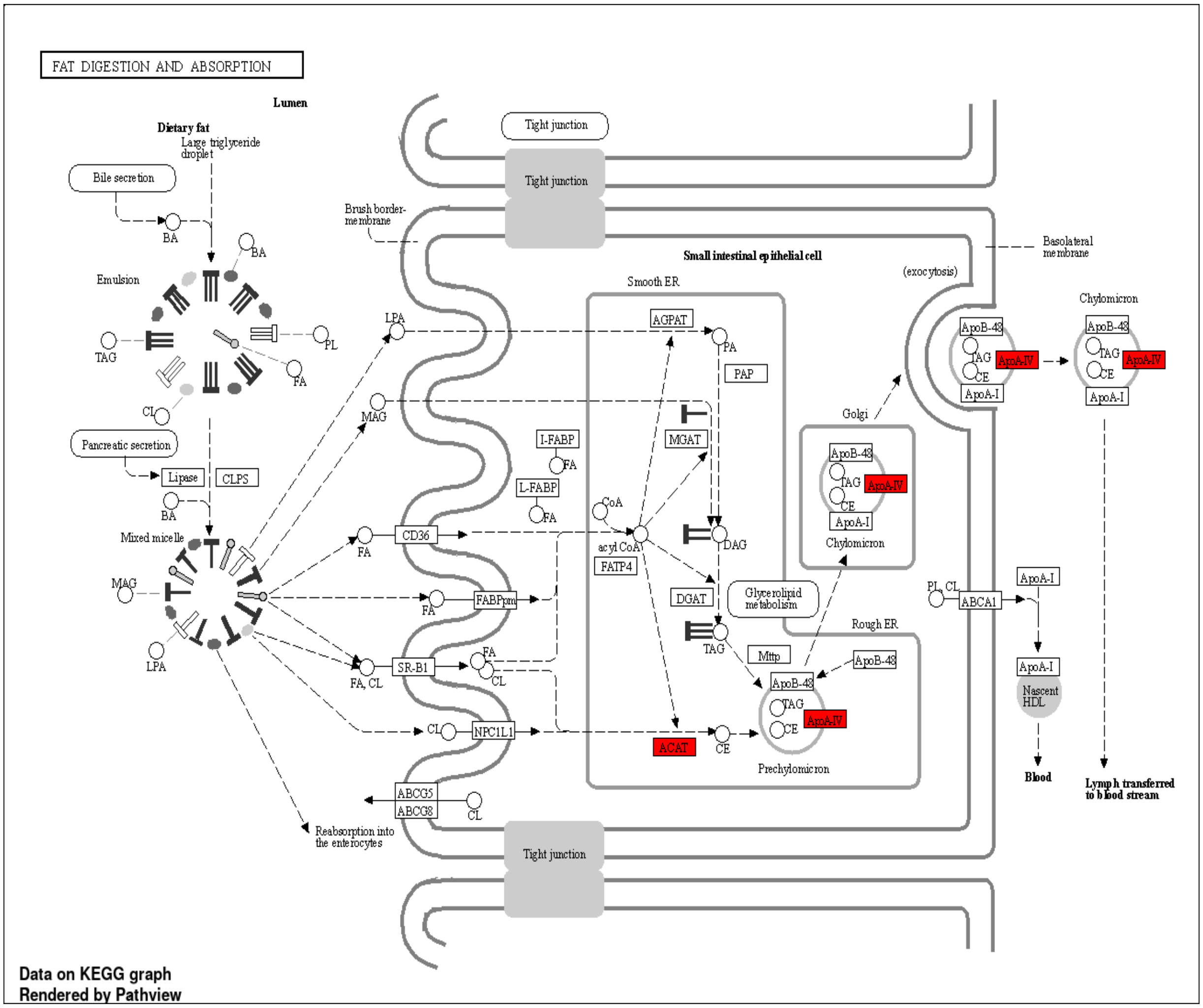

Figure S9: Fat digestion and absorption, Wild-type samples. The highlighted ACAT representing Acetyl-CoA acetyltransferase, mitochondrial and ApoA-IV representing Apolipoprotein A-IV, the two unique identifier proteins appear only in Wild-type group and are involved in fat digestion and absorption.

Figure S10A-B

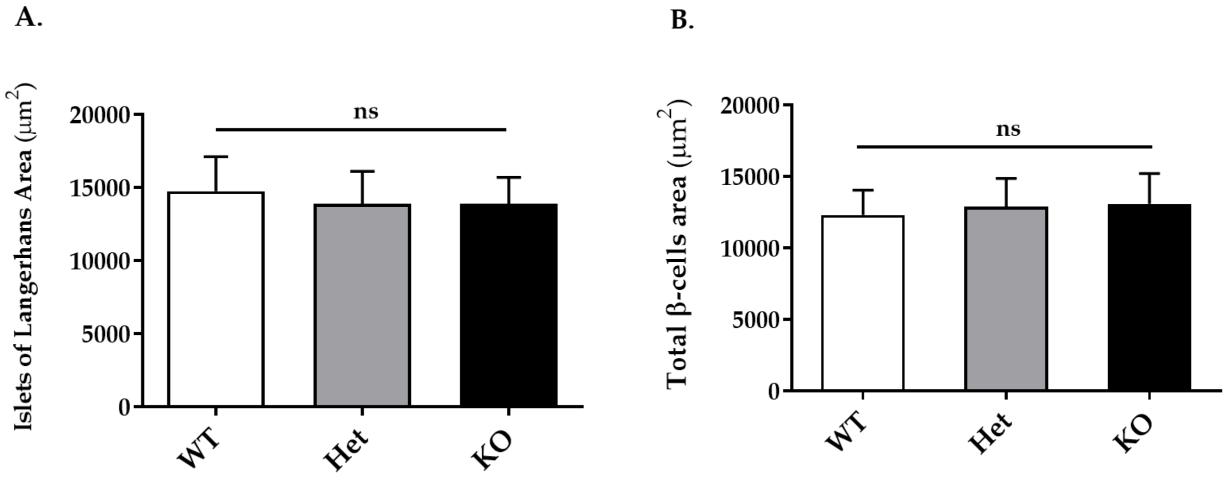

Figure S10: Islets area and β-cells total area measurement. (**A**) The area of islets measured in H&E stained sections for Wild-type (+/+) (WT, white bar, n=43 islets, 3 mice), *Rap1A* heterozygous (−/+) (Het, gray bar, n=43 islets, 3 mice), and *Rap1A* knock-out (−/−) (KO, black bar, n=60 islets, 3 mice). (**B**) The total β-cells area, measured using anti-insulin stained section's images, for WT (white bar, n=32, 3 mice), Het (gray bar, n=28, 3 mice), and KO (black bar, n=27, 3 mice); ns, not significant.

Figure S11A-B

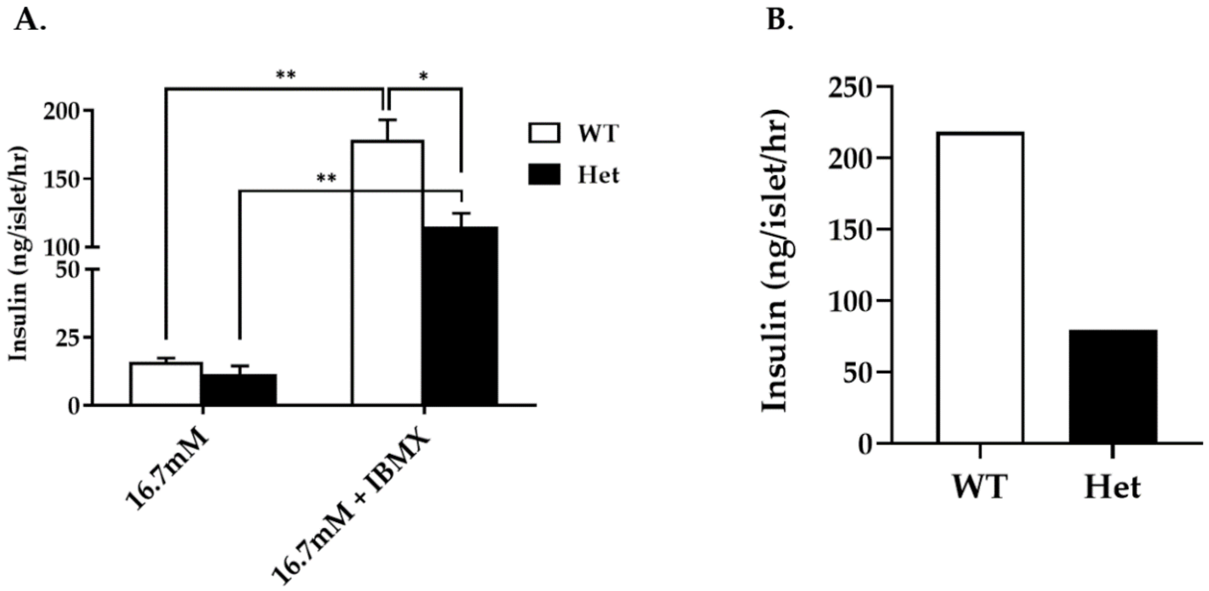

Figure S11: Preliminary Studies assessing the *ex vivo* insulin secretion from isolated islets of Langerhans of Wild-type (WT) and *Rap1A*-heterozygous $^{(-/+)}$ (Het) C57BL/6 mice in (**A**) stimulatory glucose concentration of 16.7 mM alone, or in combination with 3-isobutyl-1-methylxanthine (IBMX, 0.1 mM), an inhibitor of cyclic nucleotide phosphodiesterases, which can increase intracellular levels of cAMP. Insulin secretion increased about 10-fold with addition of IBMX (** $p<0.001$), however, insulin secretion was decreased in *Rap1A* Het as compared to control WT mice in presence of IBMX (*, $p<0.05$, n=2 independent replicates, with 2 islets each/replicate). (**B**) Similarly, Rap1A-Wild-type $^{(+/+)}$ (WT) and –heterozygous $^{(-/+)}$ (Het) islets were incubated with the adenylyl cyclase activator and cAMP elevating agent forskolin along with glucose (16.7 mM), which showed reduced insulin secretion in *Rap1A* Het compared to WT (n=1, with 2 islets each/replicate).

Supplementary Table S1: Densitometry analysis of 1D-SDS-PAGE (Table S3)

Table S1: Differential Expression levels after log2 Fold change semi-quantification of 1D-SDS-PAGE.

WT Band Numbers	Corresponding band Numbers	WT BAND %			Mean %	NULL MICE BAND %			Mean %	log2 fold Change	Protein Expression	P-value	Significant OR Non-significant
		WT 1	WT 2	WT 3		Null 1	Null 2	Null 3					
**Band 1**	Null Band 1	1.95	0.614	1.595	4.167	0	0.12	0.64	0.776	-2.424	Down regulated	0.121	Non-significant
**Band 2+4**	Null Band 2	3.69	3.995	4.287	11.982	2.36	1.81	1.45	5.635	-1.088	Down regulated	0.039	Significant
**Band 6**	Null Band 3	8.59	8.401	8.374	25.371	7.72	8.29	6.63	22.645	-0.164	Down regulated	0.193	Non-significant
**Band 7**	Null Band 4	26.5	28.17	26.42	81.146	36.1	35.8	36.8	108.88	0.424	Up regulated	0.007	Significant
**Band 8**	Null Band 5	3.48	5.02	4.287	12.789	3.113	2.72	2.75	8.583	-0.575	Down regulated	0.129	Non-significant
**Band 9**	Null Band 6	15.0	13.83	12.66	41.509	5.97	6.08	5.50	17.566	-1.240	Down regulated	0.004	Significant
**Band 10**	Null Band 7	5.44	5.02	5.882	16.342	4.10	4.40	4.20	12.72	-0.361	Down regulated	0.060	Non-significant
**Band 11**	Null Band 8	4.35	4.405	4.386	13.143	3.11	3.88	3.236	10.226	-0.362	Down regulated	0.049	Significant
**Band 12**	Null Band 9	5.22	4.918	3.788	13.929	11.95	10.10	12.45	34.517	1.309	Up regulated	0.020	Significant
**Band 13**	Null Band 11	12.0	11.98	14.75	38.82	7.59	7.25	6.14	20.997	-0.886	Down regulated	0.046	Significant
**Band 14**	Null Band 12	5.00	5.122	5.483	15.61	2.86	2.97	3.07	8.917	-0.807	Down regulated	0.001	Significant

Supplementary Table S2

Table S2: List of proteins that were identified as uniquely expressed in Rap1A knock-out $^{(-/-)}$ mice samples using nanoLC-ESI-MS/MS.

No.	Accession	Protein	Gene symbol	MW [kDa]	pI	Scores	SC [%]	# Peptides
1	ERO1B_MOUSE	ERO1-like protein beta	ERO1B	53.5	8.4	76.6	7.3	3
2	TBA1B_MOUSE	Tubulin alpha-1B chain	Tuba1b	50.1	4.9	16.3	10.4	3
3	ACTC_MOUSE	Actin, alpha cardiac muscle 1	Actc1	42.0	5.2	502.5	13.0	3
4	TPIS_MOUSE	Triosephosphate isomerase	Tpi1	32.2	5.6	84.6	9.7	2
5	1433G_MOUSE	14-3-3 protein gamma	Ywhag	28.3	4.8	32.0	8.9	2
6	VIGLN_MOUSE	Vigilin	Hdlbp	141.7	6.4	28.4	3.8	4
7	KLK1_MOUSE	Kallikrein-1	KLK1	28.8	4.9	96.5	16.1	4
8	RACK1_MOUSE	Receptor of activated protein C kinase 1	RACK1	35.1	7.6	108.4	12.3	3

Supplementary Table S3

Table S3: List of proteins that were identified as uniquely expressed in Rap1A Wild-type using nanoLC-ESI-MS/MS.

No.	Accession	Protein	Gene symbol	MW [kDa]	pI	Scores	SC [%]	# Peptides
1	EWS_MOUSE	RNA-binding protein EWS	Ewsr1	68.4	9.4	175.1	8.1	4
2	RRBP1_MOUSE	Ribosome-binding protein 1	Rrbp1	172.8	9.4	78.4	1.9	2
3	HNRPQ_MOUSE	Heterogeneous nuclear ribonucleoprotein Q	Syncrip	69.6	8.7	115.9	7.1	4
4	CAVN2_MOUSE	Caveolae-associated protein 2	Sdpr	46.7	5.2	85.8	13.6	4
5	NUCB2_MOUSE	Nucleobindin-2	Nucb2	50.3	5.0	139.7	9.8	3
6	OST48_MOUSE	Dolichyl-diphosphooligosaccharide--protein glycosyltransferase 48 kDa subunit	Ddost	49.0	5.5	21.6	7.5	3
7	CYB5_MOUSE	Cytochrome b5	Cyb5b	15.2	4.9	47.1	34.3	3
8	H2B1B_MOUSE	Histone H2B type 1-B	Hist1h2bb	13.9	10.3	32.0	19.0	2
9	NUCL_MOUSE	Nucleolin	Ncl	76.7	4.7	99.6	9.3	5
10	CUZD1_MOUSE	CUB and zona pellucida-like domain-containing protein 1	Cuzd1	68.0	6.2	109.2	4.6	2
11	SFPQ_MOUSE	Splicing factor, proline- and glutamine-rich	Sfpq	75.4	9.5	156.6	7.7	4
12	CALX_MOUSE	Calnexin		67.2	4.5	167.9	7.4	3
13	EZRI_MOUSE	Ezrin	Ezr	69.4	5.8	129.8	3.2	2
14	GP2_MOUSE	Pancreatic secretory granule membrane major glycoprotein GP2	Gp2	59.1	5.0	135.2	4.0	2
15	FUBP1_MOUSE	Far upstream element-binding protein 1	Fubp1	68.5	7.7	86.9	4.0	2
16	TRFE_MOUSE	Serotransferrin	Trf	76.7	6.9	29.7	3.4	2
17	LMNA_MOUSE	Prelamin-A/C	Lmna	74.2	6.5	22.1	3.5	3
18	PRRC1_MOUSE	Protein PRRC1	Prrc1	46.3	5.6	142.7	20.3	5
19	SBP1_MOUSE	Methanethiol oxidase	Selenbp1	52.5	5.9	30.3	10.6	4
20	ODO2_MOUSE	Dihydrolipoyllysine-residue succinyltransferase component of	Dlst	49.0	9.1	116.1	13.0	6

		2-oxoglutarate dehydrogenase complex, mitochondrial						
21	DCTN2_MOUSE	Dynactin subunit 2	Dctn2	44.1	5.1	68.8	18.2	5
22	F10A1_MOUSE	Hsc70-interacting protein	St13	41.6	5.2	26.9	13.2	4
23	FUMH_MOUSE	Fumarate hydratase, mitochondrial	Fh1	54.3	9.1	41.8	6.1	3
24	APOA4_MOUSE	Apolipoprotein A-IV	Apoa4	45.0	5.3	165.4	26.8	8
25	NSF1C_MOUSE	NSFL1 cofactor p47	Nsfl1c<br>Suclg2	40.7	5.0	323.1	22.7	6
26	SUCB2_MOUSE	Succinate--CoA ligase [GDP-forming] subunit beta, mitochondrial	Suclg2	46.8	6.6	18.6	13.2	5
27	THIL_MOUSE	Acetyl-CoA acetyltransferase, mitochondrial	Acat1	44.8	8.7	21.9	5.9	2
28	PURA_MOUSE	Transcriptional activator protein Pur-alpha	Pura	34.9	6.1	25.7	10.0	2
29	TPM1_MOUSE	Tropomyosin alpha-1 chain	Tpm1	32.7	4.7	113.4	8.5	3
30	TPM2_MOUSE	Tropomyosin beta chain	Tpm2	32.8	4.7	111.0	3.9	2
31	HTRA2_MOUSE	Serine protease HTRA2, mitochondrial	Htra2	49.3	9.6	15.1	5.5	2
32	TAGL_MOUSE	Transgelin	Tagln	22.6	8.9	117.0	20.4	6
33	KCY_MOUSE	UMP-CMP kinase	Cmpk1	22.2	5.7	92.0	30.6	5
34	TAGL2_MOUSE	Transgelin-2	Tagln2	22.4	8.4	144.6	27.1	5
35	TCTP_MOUSE	Translationally-controlled tumor protein	Tpt1	19.4	4.8	268.6	51.7	5
36	RAB1A_MOUSE	Ras-related protein Rab-1A	*Rab1a*	22.7	5.9	82.5	38.5	5
37	RAB2A_MOUSE	Ras-related protein Rab-2A	Rab2a	23.5	6.1	61.0	24.1	4
38	TMEDA_MOUSE	Transmembrane emp24 domain-containing protein 10	Tmed10	24.9	6.2	130.0	18.7	4
39	BTF3_MOUSE	Transcription factor BTF3	Btf3	22.0	9.5	276.6	48.0	4
40	PRDX3_MOUSE	Thioredoxin-dependent peroxide reductase, mitochondrial	Prdx3	28.1	7.2	56.0	14.4	3
41	BT3L4_MOUSE	Transcription factor BTF3 homolog 4	Btf3l4	17.3	6.0	19.1	36.7	3

42	PEBP1_MOUSE	Phosphatidylethanolamine-binding protein 1	Pebp1	20.8	5.2	66.7	28.3	3
43	ABHEB_MOUSE	Protein ABHD14B	Abhd14b	22.4	5.8	66.9	10.0	2
44	FRIH_MOUSE	Ferritin heavy chain	Fth1	21.1	5.5	165.1	9.9	2
45	RAB7A_MOUSE	Ras-related protein Rab-7a	Rab7	23.5	6.4	16.5	11.6	2
46	CRK_MOUSE	Adapter molecule crk	Crk	33.8	5.4	20.6	10.9	2
47	FABP4_MOUSE	Fatty acid-binding protein, adipocyte	Fabp4	14.6	8.5	440.5	53.0	5
48	NDUA6_MOUSE	NADH dehydrogenase [ubiquinone] 1 alpha subcomplex subunit 6	Ndufa6	15.3	10.1	146.0	19.1	2
49	TTHY_MOUSE	Transthyretin	Ttr	15.8	5.8	94.4	24.5	2
50	TM263_MOUSE	Transmembrane protein 263	Tmem263	11.5	9.3	22.5	36.5	2
51	HINT1_MOUSE	Histidine triad nucleotide-binding protein 1	Hint1	13.8	6.4	150.8	30.2	2
52	ACYP1_MOUSE	Acylphosphatase-1	Acyp1	11.2	9.0	98.4	35.4	3
53	DOPD_MOUSE	D-dopachrome decarboxylase	Ddt	13.1	6.1	87.0	39.0	3
54	ACBP_MOUSE	Acyl-CoA-binding protein	Dbi	10.0	8.8	223.5	39.1	2
55	DLRB1_MOUSE	Dynein light chain roadblock-type 1	Dynlrb1	11.0	6.6	234.8	29.2	2
56	YBOX3_MOUSE	Y-box-binding protein 3	Ybx3	38.8	9.7	57.2	21.9	3

Supplementary Table S4: Overlapping proteins expressed in both Rap1A Wild-type and Rap1A Knock-out $^{(-/-)}$

Table S4: List of overlapping proteins expressed in both Rap1A Wild-type and Rap1A Knock-out samples identified using NanoLC-ESI-MS/MS.

No.	Accession	Protein	MW [kDa]	pI	Scores	SC [%]	#Peptides
1	AMYP_MOUSE	Pancreatic alpha-amylase	57.3	6.9	6349.0	42.3	18
2	BIP_MOUSE	Endoplasmic reticulum chaperone BiP	72.4	5.1	1372.7	28.4	15
3	CBPA1_MOUSE	Carboxypeptidase A1	47.4	5.4	106.8	17.4	6
4	LIPP_MOUSE	Pancreatic triacylglycerol lipase	51.4	6.4	585.2	22.4	6
5	ALBU_MOUSE	Serum albumin	68.6	5.7	351.1	11.2	5
6	CEL_MOUSE	Bile salt-activated lipase	65.8	5.9	193.2	9.5	5
7	PDIA2_MOUSE	Protein disulfide-isomerase A2	58.3	4.9	280.0	12.5	5
8	ATPB_MOUSE	ATP synthase subunit beta, mitochondrial	56.3	5.2	83.7	11.0	4
9	ENPL_MOUSE	Endoplasmin	92.4	4.7	289.6	4.2	3
10	PDIA1_MOUSE	Protein disulfide-isomerase	57.0	4.8	151.8	6.3	2
11	GSTP1_MOUSE	Glutathione S-transferase	23.6	7.7	391.5	12.9	2
12	HYOU1_MOUSE	Hypoxia up-regulated protein 1	111.1	5.1	481.7	17.9	12
13	HBB1_MOUSE	Hemoglobin subunit beta-1	15.8	7.1	18.9	38.8	4
14	CTRB1_MOUSE	Chymotrypsinogen B	27.8	4.9	89.6	15.2	3
15	HBA_MOUSE	Hemoglobin subunit alpha	15.1	8.0	40.8	28.2	3
16	CELA1_MOUSE	Chymotrypsin-like elastase family member 1	28.9	8.6	97.6	18.8	3
17	EF1A1_MOUSE	Elongation factor 1-alpha 1	50.1	9.1	70.4	5.0	2
18	ACTB_MOUSE	Actin, cytoplasmic 1	41.7	5.3	23.3	9.1	2
19	RSSA_MOUSE	40S ribosomal protein SA	32.8	4.8	73.1	10.2	2
20	CEL2A_MOUSE	Chymotrypsin-like elastase family member 2A	28.9	9.0	114.2	13.3	2
21	PDIA4_MOUSE	Protein disulfide-isomerase A4	71.9	5.2	35.3	5.8	3
22	TRY2_MOUSE	Anionic trypsin-2	26.2	4.4	357.3	12.2	2
23	ATPA_MOUSE	ATP synthase subunit alpha, mitochondrial	59.7	9.2	1255.6	39.6	18
24	A1AT1_MOUSE	Alpha-1-antitrypsin 1-1	46.0	5.4	545.4	28.3	10
25	PDIA3_MOUSE	Protein disulfide-isomerase A3	56.6	5.9	127.7	23.2	9
26	EF1A1_MOUSE	Elongation factor 1-alpha 1	50.1	9.1	110.7	6.7	3
27	SAHH_MOUSE	Adenosylhomocysteinase	47.7	6.1	96.3	13.0	5
28	EF1B_MOUSE	Elongation factor 1-beta	24.7	4.5	106.5	12.4	2
29	EF1D_MOUSE	Elongation factor 1-delta	31.3	4.9	252.8	8.9	2
30	ATPO_MOUSE	ATP synthase subunit O, mitochondrial	23.3	10.0	198.8	33.3	5
31	ATP5I_MOUSE	ATP synthase subunit e, mitochondrial	8.2	9.3	137.8	50.7	3
32	CALR_MOUSE	Calreticulin	48.0	4.3	2316.8	43.5	16
33	SPI2_MOUSE	Serpin I2	45.7	5.8	43.8	13.1	5
34	PPIA_MOUSE	Peptidyl-prolyl cis-trans isomerase A	18.0	7.7	1234.0	60.4	8
35	RL38_MOUSE	60S ribosomal protein L38	8.2	10.1	578.5	50.0	4

36	SODC_MOUSE	Superoxide dismutase [Cu-Zn]	15.9	6.0	1053.7	39.0	6
37	ACTBL_MOUSE	Beta-actin-like protein 2	42.0	5.3	14.5	9.0	2
38	HSP7C_MOUSE	Heat shock cognate 71 kDa protein	70.8	5.4	431.7	13.6	6
39	ILEUA_MOUSE	Leukocyte elastase inhibitor A	42.5	5.9	234.3	21.9	7
40	VIME_MOUSE	Vimentin	53.7	5.1	1370.4	38.2	19
41	DESM_MOUSE	Desmin	53.5	5.2	345.2	17.9	7
42	SPTCS_MOUSE	Spatacsin	273.8	5.6	23.9	0.7	2
43	IF4A1_MOUSE	Eukaryotic initiation factor 4A-I	46.1	5.3	213.2	19.5	6
44	DJB11_MOUSE	DnaJ homolog subfamily B member 11	40.5	5.9	589.6	27.9	11
45	ACTA_MOUSE	Actin, aortic smooth muscle	42.0	5.2	202.2	10.3	2
46	ANXA5_MOUSE	Annexin A5	35.7	4.8	540.1	23.8	6
47	MDHC_MOUSE	Malate dehydrogenase, cytoplasmic	36.5	6.2	26.8	7.5	2
48	PRDX4_MOUSE	Peroxiredoxin-4	31.0	6.7	186.5	18.2	4
49	GDIR1_MOUSE	Rho GDP-dissociation inhibitor 1	23.4	5.1	38.5	15.2	2
50	FRIL1_MOUSE	Ferritin light chain 1	20.8	5.7	1024.2	44.8	6
51	HEMO_MOUSE	Hemopexin	51.3	7.9	21.1	8.5	3
52	COL_MOUSE	Colipase	12.4	8.0	201.3	34.5	4
53	HSP7C_MOUSE	Heat shock cognate 71 kDa protein	70.8	5.4	431.7	13.6	6
54	IF4A1_MOUSE	Eukaryotic initiation factor 4A-I	46.1	5.3	213.2	19.5	6
55	CH10_MOUSE	10 kDa heat shock protein, mitochondrial	11.0	7.9	4171.6	47.1	6
56	RS27A_MOUSE	Ubiquitin-40S ribosomal protein S27a	17.9	9.7	35.0	16.0	2
57	TRY2_MOUSE	Anionic trypsin-2	26.2	4.4	272.8	12.2	2
58	LIPR1_MOUSE	Inactive pancreatic lipase-related protein 1	52.7	5.9	715.0	29.6	8
59	SAHH_MOUSE	Adenosylhomocysteinase	47.7	6.1	96.3	13.0	5
60	CREL2_MOUSE	Protein disulfide isomerase Creld2	38.2	4.5	171.7	19.7	5
61	PHS_MOUSE	Pterin-4-alpha-carbinolamine dehydratase	12.0	6.3	406.7	23.1	3
62	QCR2_MOUSE	Cytochrome b-c1 complex subunit 2, mitochondrial	48.2	9.3	14.3	7.1	2
63	IGH1M_MOUSE	Ig gamma-1 chain C region, membrane-bound form	43.4	6.0	15.7	7.1	2
64	IF4B_MOUSE	Eukaryotic translation initiation factor 4B	68.8	5.5	319.2	7.2	3
65	PABP1_MOUSE	Polyadenylate-binding protein 1	70.6	9.5	84.5	10.5	5
66	ERP29_MOUSE	Endoplasmic reticulum resident protein 29	28.8	5.9	24.4	8.0	2
67	YBOX1_MOUSE	Y-box-binding protein 1	35.7	9.9	24.7	15.2	2
68	THIO_MOUSE	Thioredoxin	11.7	4.8	111.0	22.9	2
69	LEG1_MOUSE	Galectin-1	14.9	5.3	24.0	17.8	2
70	NACA_MOUSE	Nascent polypeptide-associated complex subunit alpha	23.4	4.5	237.2	25.6	4
71	ARF1_MOUSE	ADP-ribosylation factor 1	20.7	6.3	25.4	13.8	2
72	CNPY2_MOUSE	Protein canopy homolog 2	20.8	4.9	246.9	22.5	3
73	GLU2B_MOUSE	Glucosidase 2 subunit beta	58.8	4.4	155.2	5.8	3
74	ML12B_MOUSE	Myosin regulatory light chain 12B	19.8	4.7	42.7	12.2	2
75	SSRA_MOUSE	Translocon-associated protein subunit alpha	32.0	4.3	164.4	11.9	3
76	SERA_MOUSE	D-3-phosphoglycerate dehydrogenase	56.5	6.1	120.7	5.1	2

77	ENOA_MOUSE	Alpha-enolase	47.1	6.4	72.6	16.8	4

Supplementary Table S5

***Table S5*: Gene Ontology Rap1A Knock-out group: Biological Process.**

Enrichment FDR	nGenes	Pathway Genes	Fold Enrichment	Pathway	Protein Id's
0.027	1	6	1215.83	Protein maturation by protein folding	Ero1b
0.027	1	7	1042.14	Regulation of hydrogen peroxide-induced neuron death	Rack1
0.027	1	7	1042.14	Negative regulation of hydrogen peroxide-induced neuron death	Rack1
0.027	1	7	1042.14	Positive regulation of gastrulation	Rack1
0.027	1	7	1042.14	Neuron death in response to hydrogen peroxide	Rack1
0.027	1	7	1042.14	Rescue of stalled ribosome	Rack1
0.030	1	9	810.555	Regulation of cyclic-nucleotide phosphodiesterase activity	Rack1
0.030	1	10	729.5	Peptidyl-proline hydroxylation to 4-hydroxy-L-proline	Ero1b
0.030	1	10	729.5	Positive regulation of mitochondrial depolarization	Rack1
0.030	1	11	663.18	Insulin metabolic process	Ero1b
0.030	1	12	607.91	Positive regulation of membrane depolarization	Rack1
0.030	1	12	607.916	Regulation of Golgi to plasma membrane protein transport	Rack1
0.037	1	22	331.590	Signaling receptor ligand precursor processing	Ero1b
0.037	1	24	303.93	Negative regulation of oxidative stress-induced neuron death	Rack1
0.037	1	25	291.8	Negative regulation of response to reactive oxygen species	Rack1
0.027	2	229	63.71	Protein processing	Ero1b Klk1
0.027	2	289	50.48	Carbohydrate homeostasis	Rack1 Ero1b
0.027	2	289	50.48	Glucose homeostasis	Rack1 Ero1b
0.027	2	296	49.29	Protein maturation	Ero1b Klk1
0.027	3	1799	12.16	Proteolysis	Rack1 Ero1b Klk1

Supplementary Table S6

Table S6: Gene Ontology of Rap1A Knock-out mice group: Molecular function.

N	High level GO molecular function category	Protein Id's
3	Hydrolase activity	Tuba1b Klk1 Actc1
2	Lyase activity	Tpi1
2	Isomerase activity	Tpi1 Ero1b
2	Small molecule binding	Tuba1b Actc1
2	Protein-containing complex binding	Rack1 Eno1b
2	Carbohydrate derivative binding	Tuba1b Actc1
1	Structural molecule activity	Tuba1b
1	Molecular transducer activity	Rack1
1	Molecular function regulator	Rack1
1	Structural constituent of cytoskeleton	Tuba1b
1	Channel regulator activity	Rack1
1	Oxidoreductase activity	Ero1b
1	Enzyme regulator activity	Rack1
1	Signaling receptor activity	Rack1
1	Molecular adaptor activity	Rack1

Supplementary Table S7

Table S7: Gene Ontology of Rap1A Knock-out group: Cellular components.

N	High level GO cellular component category	Protein Id's
3	Cell projection	Rack1 Eno1b Actc1
3	Non-membrane-bounded organelle	Rack1 Tuba1b Actc1
2	Cell junction	Eno1b Actc1
2	Synapse	Eno1b Actc1
2	Supramolecular fiber	Tuba1b Actc1
2	Endomembrane system	Ero1b Klk1
2	Myelin sheath	Tuba1b Eno1b
2	Cell body	Rack1 Actc1
2	Polymeric cytoskeletal fiber	Tuba1b Actc1
1	Extracellular region	Klk1
1	Membrane-enclosed lumen	Rack1
1	Extracellular space	Klk1
1	Endoplasmic reticulum membrane	Ero1b
1	Midbody	Rack1
1	Organelle membrane	Ero1b
1	Cell leading edge	Actc1
1	Nuclear outer membrane-endoplasmic reticulum membrane network	Ero1b
1	Organelle lumen	Rack1
1	Glutamatergic synapse	Actc1
1	Ribonucleoprotein complex	Rack1

Supplementary Table S8

Table S8: Gene Ontology of Wild-type group: Biological Process.

Enrichment FDR	nGenes	Pathway Genes	Fold Enrichment	Pathway	Identifier Proteins
0.004	2	6	317.17	Dendritic transport of ribonucleoprotein complex	Sfpq Pura
0.017	2	18	105.72	Establishment of melanosome localization	Rab1a Dctn2
0.018	2	22	86.50	Hydrogen peroxide catabolic process	Prdx3 Apoa4
0.048	2	46	41.37	Positive regulation of fatty acid metabolic process	Nucb2 Apoa4
0.033	3	153	18.65	Transport along microtubule	Rab1a Sfpq Pura
0.004	5	276	17.23	Cellular response to oxidative stress	Prdx3 Sfpq Apoa4 Tpm1 Htra2
0.013	4	224	16.99	Response to reactive oxygen species	Crk Prdx3 Apoa4 Tpm1
0.015	4	240	15.85	Response to toxic substance	Prdx3 Apoa4 Pebp1 Htra2
0.00	7	442	15.06	Response to oxidative stress	Crk Prdx3 Sfpq Apoa4 Tpm1 Pebp1 Htra2
0.025	5	601	7.916	Response to inorganic substance	Crk Prdx3 Apoa4 Pebp1 Fabp4
0.038	6	1083	5.271	Regulation of organelle organization	Crk Lmna Sfpq Tpm1 Pebp1 Htra2
0.038	7	1524	4.370	Biological process involved in interspecies interaction between organisms	Crk Rab1a Prdx3 Sfpq Apoa4 Fabp4 Rab7

Supplementary Table S9

Table S9: Gene Ontology of Wild-type group: Molecular functions.

N	High level GO category	Identifier Proteins
6	Hydrolase activity	Acyp1 Rab1a Hint1 Rab2a Htra2 Rab7
6	Small molecule binding	Rab1a Hint1 Apoa4 Pebp1 Rab2a Rab7
5	Molecular function regulator	Prdx3 Nucb2 Apoa4 Pebp1 Rab7
4	Enzyme regulator activity	Prdx3 Nucb2 Apoa4 Pebp1
4	Protein-containing complex binding	Dctn2 Tpm2 Tpm1 Rab7
4	Carbohydrate derivative binding	Rab1a Pebp1 Rab2a Rab7
3	Structural molecule activity	Tpm2 Gp2 Tpm1
3	Lipid binding	Apoa4 Pebp1 Fabp4
2	Transporter activity	Apoa4 Fabp4
2	Antioxidant activity	Prdx3 Apoa4
2	Structural constituent of cytoskeleton	Tpm2 Tpm1
1	G protein activity	Rab7
1	Translation regulator activity	Pura
1	DNA-binding transcription factor activity	Pura
1	Antigen binding	Gp2
1	Peroxidase activity	Prdx3
1	Guanyl-nucleotide exchange factor activity	Nucb2
1	Extracellular matrix structural constituent	Gp2
1	Oxidoreductase activity	Prdx3
1	Translation repressor activity	Pura
1	Quaternary ammonium group binding	Apoa4
1	Metal cluster binding	Gp2
1	Peroxiredoxin activity	Prdx3
1	Molecular adaptor activity	Crk
1	Translation regulator activity, nucleic acid binding	Pura

Supplementary Table S10

Table S10: Gene Ontology of Wild-type group: Cellular components.

N	High level GO category	Identifier Proteins
9	Endomembrane system	Rab1a Prrc1 Prdx3 Lmna Nucb2 Gp2 Pebp1 Rab2a Rab7
9	Non-membrane-bounded organelle	Crk Btf3 Dctn2 Lmna Tpm2 Sfpq Tpm1 Htra2 Rab7
8	Organelle membrane	Rab1a Lmna Nucb2 Gp2 Pebp1 Rab2a Htra2 Rab7
7	Cell junction	Rab1a Fubp1 Apoa4 Pebp1 Pura Rab2a Rab7
7	Synapse	Rab1a Fubp1 Apoa4 Pebp1 Pura Rab2a Rab7
6	Membrane-enclosed lumen	Hint1 Fubp1 Lmna Sfpq Fabp4 Htra2
6	Organelle lumen	Hint1 Fubp1 Lmna Sfpq Fabp4 Htra2
5	Cell projection	Dctn2 Sfpq Tpm1 Pebp1 Pura
5	Cell body	Rab1a Nucb2 Pebp1 Pura Rab2a
4	Extracellular region	Nucb2 Gp2 Apoa4 Pebp1
4	Supramolecular fiber	Dctn2 Lmna Tpm2 Tpm1
4	Extracellular space	Nucb2 Gp2 Apoa4 Pebp1
4	Envelope	Lmna Nucb2 Pebp1 Htra2
4	Presynapse	Rab1a Pebp1 Rab2a Rab7
4	Polymeric cytoskeletal fiber	Dctn2 Lmna Tpm2 Tpm1
3	Synaptic vesicle	Pebp1 Rab2a Rab7
3	Cell surface	Gp2 Apoa4 Pebp1
3	Nuclear outer membrane-endoplasmic reticulum membrane network	Rab1a Nucb2 Rab2a
2	Chromatin	Sfpq Htra2
2	Endoplasmic reticulum membrane	Rab1a Rab2a
2	Outer membrane	Nucb2 Pebp1
2	Synaptic vesicle membrane	Rab2a Rab7
2	Intrinsic component of organelle membrane	Rab2a Rab7
2	Myelin sheath	Prdx3 Pebp1
2	Apical part of cell	Gp2 Pebp1
2	Side of membrane	Gp2 Htra2
2	Intrinsic component of synaptic vesicle membrane	Rab2a Rab7
2	Plasma membrane region	Gp2 Tpm1
2	Membrane protein complex	Htra2 Rab7
1	Nuclear outer membrane	Nucb2
1	Extrinsic component of membrane	Rab7
1	Site of polarized growth	Dctn2

1	Retromer complex	Rab7
1	Cell leading edge	Tpm1
1	Protein-lipid complex	Apoa4
1	Plasma lipoprotein particle	Apoa4
1	Postsynapse	Pura
1	Glutamatergic synapse	Pura
1	Neuron to neuron synapse	Pura
1	Postsynaptic specialization	Pura
1	Ribonucleoprotein complex	Btf3

Figure 7A

mRNA expression of ERO1- β

	Rap1A-WT (n=9)	Rap1A-KO (n=9)
	1.092748	0.9286897
	1.039412	1.383426
	0.6193979	0.8264111
	1.018313	1.016195
	0.922666	1.383426
	0.96635	1.003531
	1.008054	1.051776
	0.9738173	1.253603
	0.7033696	1.214343
**AV**	**0.927125311**	**1.117933422**
SDEV	0.159454227	0.199150551
**SEM**	0.053	0.066

Figure 7B

mRNA expression of TPI-1

	Rap1A-WT (n=9)	Rap1A-KO (n=9)
	1.125412	1.063941
	0.8249239	0.9662064
	0.6828298	1.132136
	1.051672	1.222036
	0.8378251	1.300153
	0.8607134	1.289785
	0.9937652	1.003892
	0.6606437	1.178904
	1.020841	0.8522437
**AV**	**0.8954029**	**1.112144122**
SDEV	0.162987398	0.152776189
**SEM**	0.054	0.051

Figure 7 D-E

protein expression of ERO1- β and β-actin

	KO					WT				
	KO1	KO2	KO3	KO4	KO5	WT1	WT2	WT3	WT4	WT5
Mean ERO1lβ	11040.61328	8516.2	6202.41	6838.56	8683.09	4381.23	5415.61	4646.45	8492.75	5803.63
Mean β-Actin	439079.1563	484756	345318	364484	469436	310563	523454	356359	580320	436196
Ratios	0.0251	0.0175	0.01796	0.0187	0.018	0.0141	0.0103	0.013	0.014	0.0133
Mean Ratio	**0.019452**					**0.01294**				

fold change=0.019452/0.01294=1.50

	1	2	3	4	5	Mean	SDEV	SEM
**Mean β-Actin (KO)**	439079.1563	484756	345318	364484	469436	**420614**	62567.8	27982
**Mean β-Actin (WT)**	310562.75	523454	356359	580320	436196	**441378**	112260	50205.6

Figure 8

Glycated hemoglobin (HbAIc, %)

	**Rap1A-WT (n=6)**	**Rap1A-KO (n=6)**
	4.1	4.1
	3.8	3.9
	3.8	3.8
	3.9	4.4
	4.1	4.5
	4.7	3.9
**AV (%)**	**4.066666667**	**4.1**
SD	0.338624669	0.289827535
**SEM**	0.1383	0.1183

Figure 9A

Oral Glucose Tolerance Test

		Wild-type (n=12)	Wild-type (n=12)	Wild-type (n=12)	Wild-type (n=12)	Wild-type (n=12)	Wild-type (n=12)
		**0 min**	**15 min**	**30 min**	**45 min**	**60 min**	**120 min**
		86	264	205	157	143	105
		87	231	166	127	139	102
		90	298	182	145	152	97
		118	174	122	119	113	97
		119	236	225	199	155	114
		113	265	237	169	151	113
		129	312	255	217	167	119
		86	209	105	71	72	50
		76	195	211	189	121	117
		96.6	199	147	128	119	83
		113.5	232	149	147	101	97
		100.5	178	144	125	85.5	82
**Glucose concentration**	**AV (mg/dL)**	101.2166667	232.75	179	149.4166667	126.5416667	98
	SD	16.80989067	44.86976103	47.68075655	40.10318131	29.69807029	19.39071943
	**SEM**	**4.85**	**12.95**	**13.76**	**11.58**	**8.57**	**5.6**

	Null (n=8)	Null (n=8)	Null (n=8)	Null (n=8)	Null (n=8)	Null (n=8)
	**0 min**	**15 min**	**30 min**	**45 min**	**60 min**	**120 min**
	82	360	281.5	215	138	95.5
	111.5	373	210	135	144	105.5
	134	425	202	138	147	101
	106	347.5	182.5	138	109	82.5
	100	296	295	241	186	110
	89	369	362	272	223	126
	112	302	248	245	164	120
	90	296	195	154	138.5	116
**AV (mg/dL)**	103.0625	346.0625	247	192.25	156.1875	107.0625
SD	16.63996373	45.7215466	62.0811681	56.900289	34.8813678	14.0951803
**SEM**	**5.884**	**16.167**	**21.952**	**20.12**	**12.334**	**4.984**

Figure 9B

Oral Glucose Tolerance Test

		Wild-type (n=7)	Wild-type (n=6)	Wild-type (n=7)
		**0 min**	**15 min**	**30 min**
		0.26	0.85	0.26
		0.19	0.7	0.3
		0.19	0.75	0.36
		0.87	1.42	0.3
		0.73	1.11	0.47
		0.52	0.72	0.41
		0.37		0.3
**Insulin**	**AV (ng/mL)**	0.447142857	0.925	0.342857143
	SD	0.269982363	0.285709643	0.074546246
	**SEM**	**0.102**	**0.117**	**0.028**

	Null (n=5)	Null (n=4)	Null (n=4)
	**0 min**	**15 min**	**30 min**
	0.25	0.85	0.3
	0.41	0.7	0.36
	0.3	0.8	0.36
	0.3	0.43	0.8
	0.32		
**AV (ng/mL)**	0.316	0.695	0.455
SD	0.058566202	0.18734994	0.231732605
**SEM**	**0.026**	**0.094**	**0.116**

Figure 9C

Insulin Tolerance Test

		Wild-type (n=12)	Wild-type (n=12)	Wild-type (n=12)	Wild-type (n=12)	Wild-type (n=12)
		**0 min**	**15 min**	**30 min**	**45 min**	**60 min**
		94	62	35	33	35
		135	67	89	66	71
		61	46	37	48	50
		109	57	35	25	25
		75	56	48	40	32
		78	60	36	38	39
		123	88	52	37	41
		96	67	40	27	33
		83	46	34	32	35
		100	73	46	30	22
		88	70	41	30	39
		113	95	65	37	35
**Glucose concentration**	**AV (mg/dL)**	96.25	65.58333333	46.5	36.91666667	38.08333333
	SD	21.21373912	14.84133254	16.16674477	11.09838918	12.63802583
	**SEM**	**6.124**	**4.284**	**4.667**	**3.204**	**3.648**

	Null (n=9)	Null (n=9)	Null (n=9)	Null (n=9)	Null (n=9)
	**0 min**	**15 min**	**30 min**	**45 min**	**60 min**
	95	69	43	44	58
	98	64	42	37	52
	79	56	31	41	36
	101	68	46	38	37
	54	45	25	28	25
	112	60	47	38	41
	98	74	46	34	27
	95	64	34	32	29
	85	60	37	30	25
**AV (mg/dL)**	90.77777778	62.22222222	39	35.7777778	36.666667
SD	16.65666366	8.437680039	7.71362431	5.21482928	11.905881
**SEM**	**5.552**	**2.813**	**2.571**	**1.738**	**3.97**

Figure 10C-D

Wild-type (WT): Insulin secretion (% content)					
	Baseline	3 mM	17 mM	H-89	MAY-0132
Exp-1	1.35	1.4	5.1	3.97	2.1
Exp-2	0.812	0.69	4.21	3.11	2.05
Exp-3	1.02	1.03	7	5.5	3.23
Mean	1.060667	1.04	5.436667	4.193333	2.46
SD	0.271296	0.355106	1.425143	1.210551	0.667308
SEM	0.156633	0.20502	0.822807	0.698912	0.38527

Heterozygous (HET): Insulin secretion (% content)					
	Baseline	3 mM	17 mM	H-89	MAY-0132
Exp-1	0.6	1.33	4.21	1.51	3.95
Exp-2	0.85	1.08	5.1	3.12	4.08
Exp-3	1.02	1.46	5.9	2.71	3.33
Mean	0.823333	1.29	5.07	2.446667	3.786667
SD	0.211266	0.193132	0.845399	0.83668	0.400791
SEM	0.121974	0.111505	0.488092	0.483057	0.231397

Knockout (KO): Insulin secretion (% content)					
	Baseline	3 mM	17 mM	H-89	MAY-0132
Exp-1	0.67	0.77	2.21	0.73	1.8
Exp-2	0.83	0.85	2.43	0.87	1.94
Exp-3	0.91	0.94	2.73	0.9	2.15
Mean	0.803333	0.853333	2.456667	0.833333	1.963333
SD	0.122202	0.085049	0.261024	0.090738	0.176163
SEM	0.070553	0.049103	0.150702	0.052387	0.101708

Figure S10A

Islets Area (H&E stained sections)					
WT		Het		KO	
mice tag#	area μm^2	mice tag#	area μm^2	mice tag#	area μm^2
865	3070.224	870	8298.158	670	2549.88
	3424.939		4682.059		5856.754
	7673.746		4311.403		6415.459
	69428.15		2317.637		12801.5
	4187.765		3888.13		3911.587
	39188.19		4193.597		31854.64
	3881.39		6590.938		6497.366
	22989.61		21354.58		46024.07
	2505.298		41981.85		9257.198
	5836.406		21598.1		28672.06
	9202.896		24279.13		18385.57
	2842.258		22803.38		2974.19
	4589.006		15953.11		2287
	23325.54		2850.682		8699.659
	29607.38		4817.362		34630.8
	31588.19	871	3890.722		4728.067
	2399.414		4093.286		7889.4
	3630.874		12085.72		26791.82
	2459.808		6570.979		2534.717
	7174.526		4949.294		5703.307
861	3582.274		4401.086	772	4471.33
	17026.46		10963.77		15428.75
	23107.42		6438.01		11089.87
	11250.19		59293.3		7558.142
	8312.026		9289.21		10044.91
	10129.28		39505.06		8119.44
	4985.582		35098.53		5749.315
	6993.346		16844.5		20658.5
	3360.269		3146.429		5374.642
	4364.928	855	2970.432		22799.88
	10492.68		21135.17		35440.55
	15459.85		5049.216		7456.018
	2436.221		16062.75		25232.99
	54229.31		2264.63		27964.83
765	25113.24		17577.91		5707.195
	34949.36		17964.76		7124.89
	17994.57		2408.746		17459.32
	32051.12		7575.25		82772.79
	44238.44		26560.09		3246.221
	6319.944		61224.98		18323.5
	5527.57		4309.848		30474.01
	3246.869		2815.819		37597.22
	10240.34		3203.453	853	5786.64
					5056.733
					24151.35
Mean	**14753.88**	Mean	**13897.98**		7625.534
n=43 SDEV	15558.82	n=43 SDEV	14646.74		19944.66
SEM	**2372.86**	SEM	**2233.76**		2178.317
					8401.32
					8047.642
					13311.09
					7979.602
					6454.21
					3854.434
					12131.86
					4312.31
					9996.566
					2388.398
					7293.499
					7333.416
				Mean	**13913.45**
				n=60 SDEV	13890.3
				SEM	**1792.3**

Figure S10B

Total β-cells Area (insulin stained sections)

WT mice tag#	WT area μm²	Het mice tag#	Het area μm²	KO mice tag#	KO area μm²
765	24179.73	855	34401.41	670	18575.83
	18835.42		14405.43		9593.251
	16402.31		5392.526		20325.95
	7389.662		11568.36		8124.106
	4747.118		10111		4179.989
	1653.826		10288.56		5331.744
	9938.506		4875.163		11117.61
	1682.597		1418.602		34998.48
	22931.29		1624.406	772	25137.22
	16119.26	870	22700.74		19407.73
861	2418.854		21939.98		5083.819
	5812.819		9364.378		9202.378
	10612.56		20498.83		5505.019
	22240.4		14939.77		4110.005
	12127.45		11440.57		49125.92
	15077.53		5008.392		16268.17
	6055.042		3373.358		12763.79
	6000.61		8470.138		2337.984
	2554.805	871	41352.38		30168.68
	28379.55		20549.76		8656.373
	9633.168		9915.178	853	4461.739
865	2966.674		31656.48		12814.59
	5989.464		10114.11		4701.629
	1407.586		2220.437		12738.51
	12331.44		3853.008		5911.963
	36652.56		20188.7		5090.558
	31032.85		6046.747		7830.432
	27388.89		3704.875		
	15696.5				
	5239.858			Mean	**13094.94**
	850.5648	Mean	**12907.97**	n=27 SDEV	11040.4
	10289.07	n=28 SDEV	10372.17	SEM	**2124.79**
		SEM	**1960.71**		
Mean	**12332.44**				
n=32 SDEV	9743.857				
SEM	**1721.53**				

Preliminary Studies

Insulin secretion from isolated islets

Figure S11A

			n=2		n=2	
			**WT (16.7 mM)**	**HET (16.7 mM)**	**WT (16.7 mM+IBMX)**	**HET (16.7 mM+IBMX)**
			17.43	14.55	193.29	124.81
			14.48	8.54	163.52	105.1
**insulin release**	**ng/islet/hr**	**AV**	**15.955**	**11.545**	**178.405**	**114.955**
		SD	2.085965005	4.249711755	21.05056888	13.93707466
		**SEM**	**1.475**	**3.005**	**14.887**	**9.856**

Figure S11B

		n=1	
		**WT (FSK+16.7mM)**	**HET (FSK+16.7mM)**
**insulin release**	**ng/islet/hr**	**218.645**	**79.695**
